# Effect of Operational Variables on Supercritical Foaming of Caffeic Acid-Loaded Poly(lactic acid)/Poly(butylene adipate-co-terephthalate) Blends for the Development of Sustainable Materials

**DOI:** 10.3390/polym16070948

**Published:** 2024-03-30

**Authors:** Patricia Rivera, Alejandra Torres, Julio Romero, Álvaro Alarcón, Sara Martínez, Marina P. Arrieta, Francisco Rodríguez-Mercado, María José Galotto

**Affiliations:** 1 Packaging Innovation Center (LABEN), Technology Faculty, Center for the Development of Nanoscience and Nanotechnology CEDENNA, University of Santiago de Chile (USACH), Santiago 9170201, Chile; patricia.rivera.f@usach.cl (P.R.); alvaro.alarcon@usach.cl (Á.A.); sara.martinez@usach.cl (S.M.); francisco.rodriguez.m@usach.cl (F.R.-M.); maria.galotto@usach.cl (M.J.G.); 2 Laboratory of Membrane Separation Processes (LabProSeM), Department of Chemical Engineering, Engineering Faculty, University of Santiago de Chile, Santiago 9170201, Chile; julio.romero@usach.cl; 3 Departamento de Ingeniería Química Industrial y del Medio Ambiente, Escuela Técnica Superior de Ingenieros Industriales, Universidad Politécnica de Madrid (ETSII-UPM), Calle José Gutiérrez Abascal 2, 28006 Madrid, Spain; m.arrieta@upm.es; 4 Grupo de Investigación, Polímeros, Caracterización y Aplicaciones (POLCA), 28006 Madrid, Spain

**Keywords:** PLA, PBAT, blend, active foaming, supercritical carbon dioxide

## Abstract

Expanded polystyrene will account for 5.3% of total global plastic production in 2021 and is widely used for food packaging due to its excellent moisture resistance and thermal insulation. However, some of these packages are often used only once before being discarded, generating large amounts of environmentally harmful plastic waste. A very attractive alternative to the conventional methods used for polymer processing is the use of supercritical carbon dioxide (scCO_2_) since it has mass-transfer properties adapted to the foam morphology, generating different path lengths for the diffusion of active compounds within its structure and can dissolve a wide range of organic molecules under supercritical conditions. The objective of this research was to evaluate the effect of operational variables on the process of caffeic acid (CA) impregnation and subsequent foaming of polylactic acid (PLA) as well as two PLA/poly(butylene-co-terephthalate-adipate) (PBAT) blends using scCO_2_. The results showed an increase in the degree of crystallinity of the CA-impregnated samples due to the nucleation effect of the active compound. On the other hand, SEM micrographs of both films and foams showed significant differences due to the presence of PBAT and its low miscibility with PLA. Finally, the results obtained in this work contribute to the knowledge of the important parameters to consider for the implementation of the impregnation and foaming process of PLA and PLA/PBAT blends with potential use in food packaging.

## 1. Introduction

In 2021, total global plastic production was 390.7 million tons, including 5.3% expanded polystyrene (EPS) [1], commonly used in food packaging due to its excellent moisture resistance and thermal insulation, preserving the freshness of perishable products such as seafood, fruit, and vegetables [2]. However, some of this packaging is usually used only once before being discarded. Since polystyrene is not a biodegradable polymer, it must be recycled or incinerated, which leads to a large volume of waste in landfills, so most of the polystyrene continues to reach landfills around the world, causing a large environmental impact [3]. As a result, research has recently focused on the development of new plastic materials that have a low environmental impact and reduce the burden on landfills. In this sense, biodegradable polymer foams are considered a suitable and environmentally friendly substitute for current petrochemical-based polymers. Accordingly, the global market for biodegradable plastic materials is expected to grow from USD 4587.94 million in 2019 to USD 8971.32 million by the end of 2025 [4].

In this context, one of the polymers considered a promising alternative capable of competing with EPS foams due to its low cost and processing is poly(lactic acid) (PLA), which is an aliphatic thermoplastic polyester produced from renewable resources and compostable in the environment [5]. The ester group in its molecular chain gives it good degradability, as reported in some studies under controlled composting conditions. PLA can decompose into water and carbon dioxide in less than 90 days, while microorganisms can completely assimilate the degradation products [6]. In addition, PLA production requires 25–55% less energy than petroleum-based polymers [7]. However, this material has some limitations that are important for its application and foam production, including its inherent low melt strength, slow crystallization rate, and high brittleness. Considering these disadvantages, several alternatives have been studied, such as copolymerization [8], the development of bionanocomposites [9,10], and polymer blends [11,12]. The latter is the most interesting, as it is considered to be a simple and cost-effective method for the production of materials in different fields of application. Among the polymers used, poly(butylene adipate-co-terephthalate) (PBAT) stands out because it is a commercially available aliphatic-aromatic random copolymer that has excellent mechanical properties, such as flexibility and toughness, compared to other biopolymers such as poly(ε-caprolactone) (PCL) and poly(hydroxybutyrate) (PHB), characteristics that make it a suitable candidate for blending with PLA to increase PLA crystallinity and overcome its brittleness and low toughness [13]. Furthermore, recent studies have reported that PBAT is biodegradable and biocompatible, opening up a range of applications [14,15]. Several investigations on PLA/PBAT blends have been reported. Jiang et al. prepared different PLA/PBAT blends, varying the PBAT content (5–20 wt%), showing that the blend toughness and elongation at break increased as the PBAT concentration increased, although the tensile strength and modulus decreased [16]. In another study, PLA/PBAT blends with 10–50% by weight of PBAT were analyzed, obtaining that the blend with 40% by weight of PBAT presented the best balance from the evaluation of mechanical, rheological, morphological, and thermal properties [17]. Deng and coworkers [18] showed that when the PBAT content is increased from 10 to 20% by weight, the ductility of the PLA/PBAT blend system increases dramatically from about 10 to 300%. Similarly, Nofar et al. prepared PLA/PBAT blends by an injection molding (IM) process. The results showed that PLA/PBAT blends with higher PLA proportions lead to a more homogeneous blend morphology and, therefore, the ductility increased significantly [19].

On the other hand, food preservation has been studied extensively in the food industry. One way of extending the shelf-life of food products is to use active packaging technology, where a positive interaction between the product and the packaging is achieved through the action of an active ingredient incorporated into the packaging material or as part of the polymer, thereby extending the shelf-life of the product [20]. Caffeic acid (3,4-dihydroxycinnamic acid) (CA) is an important phenolic compound with a high antioxidant capacity [21], commonly present in a wide variety of plants, fruits, and propolis samples [22]. In addition, it has been shown to exhibit various bioactivities, such as antiviral, antimicrobial, and anti-inflammatory properties [23]. In this line, as reported by Cejudo and coworkers, despite the low amount of CA impregnation in PET/PP films, it presented a high antioxidant activity comparable to films impregnated with olive leaf extract [24]. This antioxidant compound has been used by several authors with supercritical carbon dioxide (scCO_2_) [25,26] and the supercritical impregnation process to impregnate CA in multilayer PET/PP composite films [24]. Both processes aim at replacing liquid organic solvents with a supercritical fluid, which offers the advantage that the final product is completely free of any residual solvent contamination [27], which is favorable for the pharmaceutical and food industries. 

Supercritical fluid technology has emerged as a very attractive alternative to conventional methods used for polymer processing. A pure component is in a supercritical state when its temperature and pressure are above critical values. Carbon dioxide (CO_2_) is by far the most widely used compound as a supercritical fluid due to its low cost; it is considered biocompatible with the human body (non-toxic) and is currently classified as GRAS by the US Food and Drug Administration (FDA) [28] and is chemically inert. In addition, CO_2_ is capable of dissolving a wide range of organic molecules under supercritical conditions (P > Pc = 73.8 bar and T > Tc = 304.15 K); this allows processes to be carried out at temperatures close to ambient, avoiding thermal degradation of organic compounds (drugs, antimicrobials, antioxidants, etc.) [29]. One of the most important applications of scCO_2_ is the supercritical impregnation, which allows the incorporation of active compounds in polymeric matrices for different applications [30], where the influence of operating variables such as temperature, pressure, contact time, and depressurization conditions on the incorporation of the active agent has been intensively explored [31,32,33,34,35,36]. 

The use of scCO_2_ as a foaming agent to produce polymeric foams has been a growing application in recent years [9,37]. Defined as a two-phase gas-polymer system [38], polymer foams using scCO_2_ can be produced on a laboratory scale (batch process) for preliminary studies as well as on a pilot scale through supercritical continuous extrusion [39]. Therefore, porous polymers can be produced by the gas foaming method, which is mainly divided into two steps. In the first, the polymer is saturated with a supercritical gas or fluid under constant temperature and pressure conditions. The system is then brought to a supersaturated state by rapidly increasing the temperature or decreasing the pressure (pressure-induced phase separation). This causes the nucleation and growth of gas bubble cells within the polymer matrix [40].

Considering the above, the objective of this research focused on the study of the effect of operational variables on the impregnation and foaming processes of biopolyesters with antioxidant activity based on PLA as well as PLA/PBAT mixtures (using two different proportions of PLA/PBAT) with CA using scCO_2_. Both types of materials, films and foams, were fully characterized in terms of their structure and thermal properties, while the amount of CA incorporated into the polymeric structures due to the impregnation process was quantified. The antioxidant activity was verified by the DDPH inhibition assay, showing the potential of the supercritical process in the active foamed polymeric structures development.

## 2. Materials and Methods

### 2.1. Materials

Poly(lactic acid) (PLA) 2003D (specific gravity 1.24; MFR g/10 min) was purchased from Natureworks^®^ Co (Minnetonka, MN, USA). Two commercial PLA/PBAT blends (BASF, Ludwigshafen, Germany), one composed of 42% PLA and 58% PBAT (trade name: Ecovio^®^ F2224) [41] and the other with a composition of 4% PLA, 84% PBAT, and 12% inert particles/additives (trade name: Ecovio^®^ F23B1) [42], were supplied by Entec Polymers, Chile (Santiago, Chile). Caffeic acid (CA) (≥98% HPLC) and 2,2-diphenyl-1-picrylhydrazyl (DPPH) were obtained from Sigma-Aldrich (Madrid, Spain). Carbon dioxide (99.9% purity) was obtained from Linde (Santiago, Chile). Chromatographic-grade acetonitrile, absolute ethanol, and other analytical-grade reagents from Merck S.A. (Darmstadt, Germany). 

### 2.2. Extrusion of PLA and PLA/PBAT Blends 

To obtain the films, different polymeric matrices were previously dried at 60 °C for 24 h and melt extruded using a 20 mm Scientific Labtech LTE20 (Muang, Thailand) corotating laboratory twin-screw extruder. The temperature profiles of the extruder from zone 1 to zone 5 were maintained between 185 and 195 °C [35] for obtaining PLA films. For the extrusion of PLA/PBAT blends, the screw speed was set at 42 rpm, and a temperature profile between 170 and 195 °C was used [43]. Films with a thickness between 500 and 600 µm were obtained. 

### 2.3. Sequential Supercritical Impregnation and Foaming

#### 2.3.1. Supercritical Impregnation of CA in PLA and PLA Polymeric Blends

**Figure 1:**
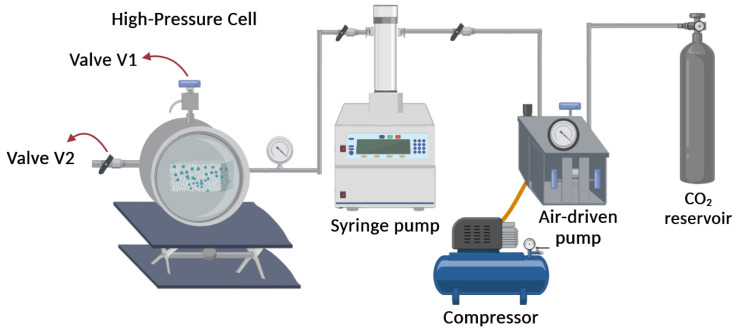
Outline of the experimental setup used for sequential supercritical impregnation and foaming processes.

The first step of the material processing involves the impregnation experiments, which were carried out in a high-pressure cell with a volume of 100 mL at a constant temperature of 40 °C using a thermostatic electrical resistance around the cell [33,35]. CO_2_ was loaded into the system using an Teledyne ISCO 500D syringe pump (Lincoln, NB, USA), operated at a constant pressure regime during each impregnation cycle. For this, plastic samples with a surface area of 6 cm^2^ and thickness between 500 and 600 µm were placed in the high-pressure cell with 10 mg of caffeic acid to ensure the saturation condition of the impregnation phase. The supercritical impregnation series were carried out at pressures of 12 and 15 MPa at a constant temperature of 40 °C for 3 h to reach equilibrium conditions. After this time, the system was depressurized at 0.1 and 1 MPa/min to subsequently characterize the obtained samples. For each impregnation condition, tests were performed in duplicate. The outline of this experimental setup is shown in Figure 1, where the micrometric valve V1 is used to depressurize the system after impregnation.

#### 2.3.2. Supercritical Foaming of Obtained Biocomposites

After impregnation, as described in Section 2.3.1, the impregnated materials were treated by supercritical foaming as follows. The foams made from PLA and PLA/PBAT blends were produced using materials with the highest active content that had been previously impregnated. The foaming process was carried out in the same experimental setup described in Figure 1. Thus, the films were placed inside the high-pressure cell, and CO_2_ was loaded into the system, which will operate at a constant pressure regime during the supercritical foaming process. The temperature inside the high-pressure cell was controlled by means of a thermocouple that was attached to the cell. Thus, the supercritical foaming was performed considering a temperature of 130 °C and pressure values of 15 and 25 MPa. The samples were kept at the selected pressure and temperature conditions for a period of approximately 25 to 30 min. The CO_2_ was then rapidly released at a depressurization rate of 60 (MPa/min) [35,44] using valve V2, and the samples were stabilized by an air-conditioned cooling system. Experiments were performed in duplicate for each condition, and the foams produced were stored in a desiccator until characterization.

### 2.4. Impregnated Films and Foams Characterization 

#### 2.4.1. Determination of the CA Content of Impregnated Films and Polymer Foams

The caffeic acid impregnated in the films and foams was quantified using the Folin–Ciocalteu method [45] with some modifications. For this purpose, a film/foam sample, 1 mL of Folin–Ciocalteu reagent, and 10 mL of distilled water were introduced into a volumetric flask and kept in the dark. After 3 min, 4 mL of 2% Na_2_CO_3_ and 10 mL of water were added to a final volume of 25 mL and kept in the dark for a minimum of 48 h. The absorbance was measured in a digital UV/VIS scanning UV/VIS spectrophotometer at 760 nm against a film/foam control sample prepared under the same reaction conditions. To determine the total phenolic content of the different films/foams, a calibration curve was prepared using standard solutions. The results were expressed as mg of caffeic acid per mg of dry film/foam [46]. All measurements were performed in duplicate.

#### 2.4.2. Thermogravimetric Analysis (TGA) 

Thermogravimetric analysis (TGA) tests were performed using a GC20 Stare System TGA/DSC, Mettler Toledo gas controller (Schwarzenbach, Switzerland). Pieces between 8 and 9 mg of films or foams were heated from 20 to 700 °C at a rate of 10 °C/min under a nitrogen atmosphere. TGA analysis allowed evaluate the thermal stability of the different PLA and PLA/PBAT blended films/foams. 

#### 2.4.3. Differential Scanning Calorimetry (DSC) 

The crystallization behavior of pure polymers, films, and foams of PLA/PBAT blends was performed using a Mettler Toledo (Columbus, OH, USA) differential scanning calorimeter (DSC) model DSC 822e. Samples (5–7 mg) were heated from −50 to 200 °C at a rate of 10 °C/min in a nitrogen atmosphere [47]. The degree of crystallinity of the films and foams was calculated by Equation (1):

(1) $$x_{c}\left(\%\right)=\frac{{∆H}_{m}-{∆H}_{cc}}{w\cdot {∆H}_{mo}}\times 100\%$$

where Δ*H_m_* is the melting enthalpy, Δ*H_cc_* is the cold-crystallization enthalpy (i.e., applicable to PLA), *w* is the weight fraction of PLA in the blend, and Δ*H_mo_* is the melting enthalpy in 100% crystalline PLA, which has a value of 93.6 J/gr [44,47].

#### 2.4.4. Attenuated Total Reflectance–Fourier-Transform Infrared (ATR-FTIR) Spectroscopy 

FTIR spectra were used to identify the presence of specific chemical groups in the films and foams developed. For this purpose, an ALPHA spectrometer equipped with an attenuated total reflection diamond crystal accessory (Bruker, Platinum) was used, using OPUS v7 software, programmed to perform 64 scans per sample in a wavelength range between 4000 and 400 cm^−1^.

#### 2.4.5. Scanning Electronic Microscopy (SEM) Analysis

The morphologies of the different foam samples were analyzed by scanning electron microscopy (SEM) using a Jeol JSM-5410 Scanning Microscope (Jeol Ltd., Akiskina, Tokyo, Japan) with accelerating voltage at 20 kV. Cell size was measured using ImageJ 1.53t software and was obtained by measuring the maximum diameter of each cell. To determine the cell size distribution, the size of at least 75 cells in the central part of the cross-section of the cryo-fractured foam sample was considered based on a Gaussian distribution approximation [37,48].

The bulk density (kg/m^3^) of the pre-foamed (*ρ_p_*) and post-foamed (*ρ_f_*) samples was determined using a pycnometer by the water displacement method according to ASTM D792-0022 [49]. Cell densities (NC) were calculated using Equation (2) [50]:

(2) $$NC=\frac{1-\frac{\rho_{f}}{\rho_{p}}}{10^{-4\;}xd^{3}}$$

Meanwhile, the expansion coefficient (ER) of the foamed samples was obtained by Equation (3) [44]:

(3) $$ER=\frac{\rho_{p}}{\rho_{f}}$$

### 2.5. Antioxidant Activity of the Obtained Films and Foams

The antioxidant effect of the obtained materials (films or foams) was measured using the DDPH inhibition assay proposed by Cejudo Bastante and coworkers [24] with some modifications. A sample of film or foam with and without active ingredient was placed in 4 mL of 6 × 10^−5^ mol/L DPPH ethanol solution. Subsequently, the absorbance was measured in a UV–visible spectrophotometer (UV-1601, Rayleigh, Beijing, China) at 515 nm at 24, 96, and 120 h of reaction in the dark at 4 °C. All analyses were performed in duplicate, and the radical scavenging activity was expressed as percentage inhibition per mass of virgin polymer, according to Equation (4):

(4) $$RSA=\frac{A_{0}-A_{i}}{A_{0}}\times 100$$

where *A_i_* and *A*_0_ represent the absorbance of the DPPH solution of the material with and without the addition of an active agent, respectively.

## 3. Results and Discussion

### 3.1. Quantification of Caffeic Acid in Impregnated Films

**Figure 2:**
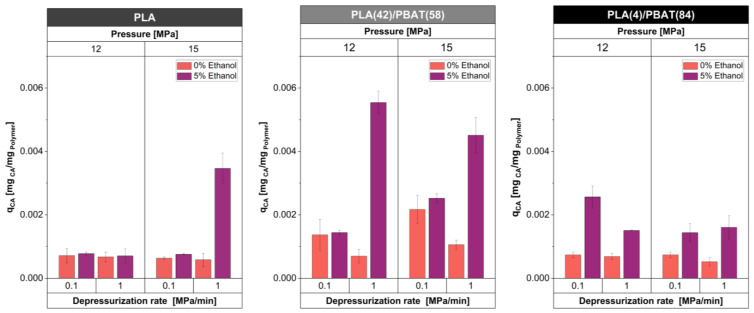
Amount of impregnated caffeic acid (q_CA_) expressed as % mg caffeic acid/mg polymer in PLA films and blends obtained at different impregnation conditions.

To determine the best impregnation conditions for each material, i.e., PLA, PLA(42)/PBAT(58), and PLA(4)/PBAT(84), the amount of caffeic acid impregnated in the different films was quantified. The materials with the highest percentage of impregnation were selected for the next foaming step. Figure 2 shows the results of the CA amount impregnated in PLA films and PLA/PBAT blends, obtained under various operating conditions, including different pressures (12 and 15 MPa), the presence of ethanol as a co-solvent (0 and 5 wt%), and varied depressurization rates (0.1 and 1 MPa/min).

Figure 2 shows that the amount of CA incorporated into the polymer matrix was improved in most cases by the use of 5 wt% ethanol solution as a co-solvent. The addition of small amounts of a co-solvent that is soluble in scCO_2_ (such as ethanol) increases the plasticizing effect of the CO_2_ on the polymer. Moreover, it increases the solubility of the active compound in the supercritical phase because ethanol increases the polarity of the high-pressure phase and, therefore, its solvating power [51,52].

In the case of the PLA-impregnated films, it was observed that the increase in the amount of CA incorporated into the polymer was mainly due to the effect of depressurization of the system. This phenomenon, known as solute deposition, occurs when CO_2_ exits the polymer matrix, causing some of the solute to recrystallize and become trapped within the polymer matrix [52]. On the other hand, the incorporation of the co-solvent ethanol into the system improved the incorporation of CA at 12 MPa by 7.9% in the PLA(42)/PBAT(58) blend and 3.5% in the PLA(4)/PBAT(84) blend.

For the blend with a higher content of PBAT, better results were obtained when the affinity of the polymeric matrix with the active compound was favored by a slow decompression at a lower pressure. However, for the PLA(42)/PBAT(58) blend, the depressurization rate of 1 MPa/min improved the amount of CA at the two different pressures used in this work (12 MPa and 15 MPa), with a higher active concentration being obtained at 12 MPa. These results account for the influence on the selection of the depressurization rate since it must be in accordance with the affinity of the active substance for the porous matrix. If the materials have a high affinity, a slow depressurization is appropriate, whereas if they have a low affinity, the active substance can be easily entrained from the matrix by the scCO_2_, and in this case, a high depressurization rate favors the entrapment of the substance in the polymer [24]. This can be explained by the fact that the CO_2_/CA partitioning has a stronger influence than the matrix/CA interaction [27]. Furthermore, the results obtained in this work are in agreement with those reported by Cejudo and coworkers [24], who impregnated PET/PP films with caffeic acid and olive leaf extract, obtaining a 6% higher impregnation compared to that obtained in this work using the following operating conditions: 40 MPa pressure, 35 °C, a depressurization rate of 10 MPa/min, and a processing time of 22 hours. Considering the above, Table 1 shows the most effective processing conditions for developed materials. These conditions were used for the foam formation, as described in Section 2.3.1.

### 3.2. Quantification of CA in Developed Foams

In order to evaluate the effect of the supercritical foaming process on the active impregnated films, the quantification of the caffeic acid in the foams was carried out. The results are presented in Table 2.

The quantified amount of CA after the foaming process was lower with increasing pressure, as can be seen for all samples. This could be because the amount of CA impregnating the films before foaming is not sufficient to saturate the system. While the material is in contact with the scCO_2_, part of the impregnated compound dissolves in the supercritical phase, which, in combination with a fast depressurization rate, causes part of the CA present in the polymer to be entrained during the foaming process. On the other hand, at 25 MPa, there is a higher loss of active compound during processing, probably because the CO_2_ density increases with increasing pressure, which is directly correlated with a higher solvent capacity of CO_2_, favoring the solubility of the active compound previously retained in the impregnated polymer [53].

### 3.3. Thermal Properties of Obtained Impregnated Films and Foams

#### 3.3.1. Thermogravimetric Analysis (TGA)

**Figure 3:**
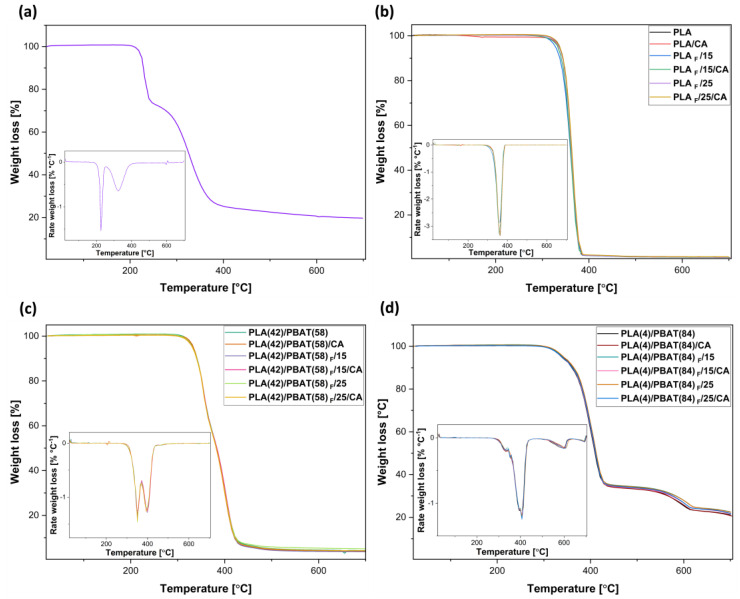
TG/DTG curves of (**a**) caffeic acid, (**b**) PLA, (**c**) PLA(42)/PBAT(58), and (**d**) PLA(4)/PBAT(84) films and foams with and without the presence of caffeic acid.

The thermal stability of the obtained materials was determined by thermogravimetric analysis. Figure 3 shows the thermograms (TG and DTG) of pure CA and the obtained impregnated films and foams. Figure 3a shows that the CA is stable above 150 °C and exhibits a two-step thermal decomposition process. The first, involving a 20% weight loss, combines melting and degradation of CA at a temperature of 228 °C, and the second can be attributed to acid decarboxylation (335 °C) with approximately 60% weight loss [22].

Regarding the results obtained for PLA samples with and without the presence of CA (Figure 3b), it can be observed that the polymer degradation occurred in only one stage, corresponding to PLA at a temperature of 363 °C, which agrees with that reported by Villegas et al. [54]. Moreover, it should be noted that the presence of impregnated CA did not affect the thermal stability of the polymer, which could be due to the low concentration of the compound in the polymeric matrix. On the other hand, the films and foams obtained from the PLA (42)/PBAT(58) blend presented thermal degradation in two stages. The first stage shows a maximum degradation temperature of 356.34 °C corresponding to the PLA fraction, and the second stage at 401 °C belonging to the PBAT part forming the blend, similar to the results obtained by Fernandes and collaborators [55], who reported the effect of nitrile rubber incorporation on commercial PLA/PBAT blends.

Otherwise, it was observed that caffeic acid incorporation by supercritical impregnation caused differences in PBAT degradation for PLA(42)/PBAT(58) films, which could be related to the fact that CA was incorporated in higher amounts in these samples. On the other hand, PLA(4)/PBAT(84) samples showed a three-stage degradation. Similar to the PLA(42)/PBAT(58) sample, the first two stages correspond to PLA (324 °C) and PBAT (403 °C). However, in this case, there is an additional stage that could correspond to the decomposition of CaCO_3_ (586 °C) into carbon dioxide and calcium oxide [56]. Taking this into account, it can be observed that the presence of CaCO_3_ reduces the thermal stability of the PLA/PBAT blend due to its catalytic effect in the depolymerization of the ester bond. This is explained by the fact that the presence of a metal ion favors the formation of free radicals and reactive terminal groups during the degradation of polyesters [56,57,58]. This is in agreement with the composition of this commercial blend (4% PLA, 84% PBAT, 12% inert particles) reported by some authors [59,60]. 

Finally, the results obtained for the foams of the different materials show that there is no significant variation in the thermal stability of the materials with or without the presence of CA. This confirms that the supercritical reprocessing of the already impregnated samples to obtain active foams does not affect their thermal stability.

#### 3.3.2. Differential Scanning Calorimetry (DSC)

Differential Scanning Calorimetry (DSC) was used to determine the effect of the supercritical process on the transition temperatures of the samples that achieved the highest incorporation of CA in the impregnation process. Table 3 shows the results obtained for impregnated PLA films and PLA/PBAT blends, as well as films without exposure to CO_2_ or CA, in order to identify possible structural modifications caused by the impregnation of the active agent. From the results, it should be noted that the T_g_ is associated with changes in the amorphous region of the polymers, which depends on the mobility of the polymer chains; T_m_ is determined by the transition of the crystalline regions and is specific to each layer; and T_cc_ is the transition from the amorphous to the crystalline state, in which the molecules in a given phase have sufficient freedom of movement to spontaneously arrange themselves in a crystalline form [54,61].

From the results presented in the table, it can be seen that the PLA(42)/PBAT(58) film shows two glass transitions at temperatures of −30 °C and 57 °C, respectively. These are related to the T_g_ of PBAT and PLA and indicate that the blends are non-miscible and have a two-phase structure [44]. A similar situation was reported by Chiu et al. [62], where PLA/PBAT blends with different proportions were obtained by injection molding with glass transition temperatures for PBAT between −35 and −30 °C and of PLA in the range between 57 and 61 °C. In contrast, in the case of the PLA(4)/PBAT(84) blend, only the glass transition associated with PBAT (−30 °C) was observed, which could be mainly due to the low amount of PLA present in this blend (4%). In addition, there were statistically significant increases in the Tm and ΔH_m_ of the PBAT and in the X_c_ of the PLA. The increase in the crystalline fraction of PLA could be due to the combined effect of the increased amount of PBAT present in the blend (84%) and the presence of calcium carbonate (CaCO_3_), which corresponds to the remaining 12% inert matter reported for this blend. The presence of PBAT, as well as CaCO_3_, can improve the crystallization behavior of PLA [62] because the rigid CaCO_3_ particles act as a nucleating agent, which contributes to higher crystallinity. [63]. This is in agreement with that reported by Teamsinsungvon et al. [58], who prepared PLA/PBAT blends and studied the effects of PLA grafted with maleic anhydride and CaCO_3_ content on the mechanical, thermal and morphological properties of the blends. On the other hand, for the samples impregnated with CA, it could be observed that there was a significant increase in the crystallinity degree (%X_c_) for the PLA(42)/PBAT(58) sample. This would be related to the amount of impregnated CA since this mixture had a higher concentration of impregnated CA. All this would confirm the above since, with the increase in X_c_, there was also a slight increase in the ΔH_m_ of PLA. In this case, the active compound CA acts as a nucleating agent in the polymer matrix, causing an increase in the degree of crystallinity.

This result is in agreement with that reported by Dintcheva and coworkers, who studied the photo-oxidation behavior of PLA with the incorporation of different compounds, where an increase in crystallinity was obtained for PLA/quercetin and PLA/vitamin E samples due to the incorporation of these antioxidants into the PLA matrix [64]. 

Crystallization is an important property to investigate in foam formation as it affects cell growth [47]. To investigate the relationship between the cell morphology of the foams and the crystals they contain, DSC analyses were carried out without removing the thermal history of the foam and film samples. Furthermore, in order to verify the changes caused by the presence of PBAT in the blends, the %X_c_ crystallinity of PLA was calculated for all samples. This also took into account the degree of crystallinity of the films with and without CA, as shown in Table 4.

Table 4 shows that the melting temperature (T_m_), melting enthalpy (ΔH_m_), and degree of crystallinity (%X_c_) were affected by the foaming process. Moreover, in all cases, these parameters were affected compared to the unfoamed samples. This is probably due to the crystallization induced by the foaming process [65] since the absorption of more CO_2_ leads to a significant swelling of the polymer matrix and a deep plasticizing effect that favors the rearrangement of the polymer chains with lower free energy [66]. 

For the PLA foams, the cold-crystallization onset temperature was not observed for all samples. The processing temperature of the foams (130 °C) was higher than the cold-crystallization onset temperature, which could indicate that the material crystallized completely during the supercritical CO_2_ process [67]. Finally, for the case of the foams generated from the PLA blends, it was observed that PLA(42)/PBAT(58) showed a statistically significant increase in the degree of crystallinity. This was due to the nucleating effect of the active compound. On the other hand, for the PLA(4)/PBAT(84) foamed samples, there was a decrease in crystallinity with increasing system pressure. This result was obtained for samples with and without caffeic acid and, according to Ni et al., would affect cell size and give rise to microcellular foams [68].

### 3.4. Structural Properties of Developed Films and Foams

For the structural analysis of the developed films and foams, ATR-FTIR tests were performed to determine the characteristic functional groups of each polymer, identified by different bands indicating vibrations and stretching associated with their chemical bonds.

**Figure 4:**
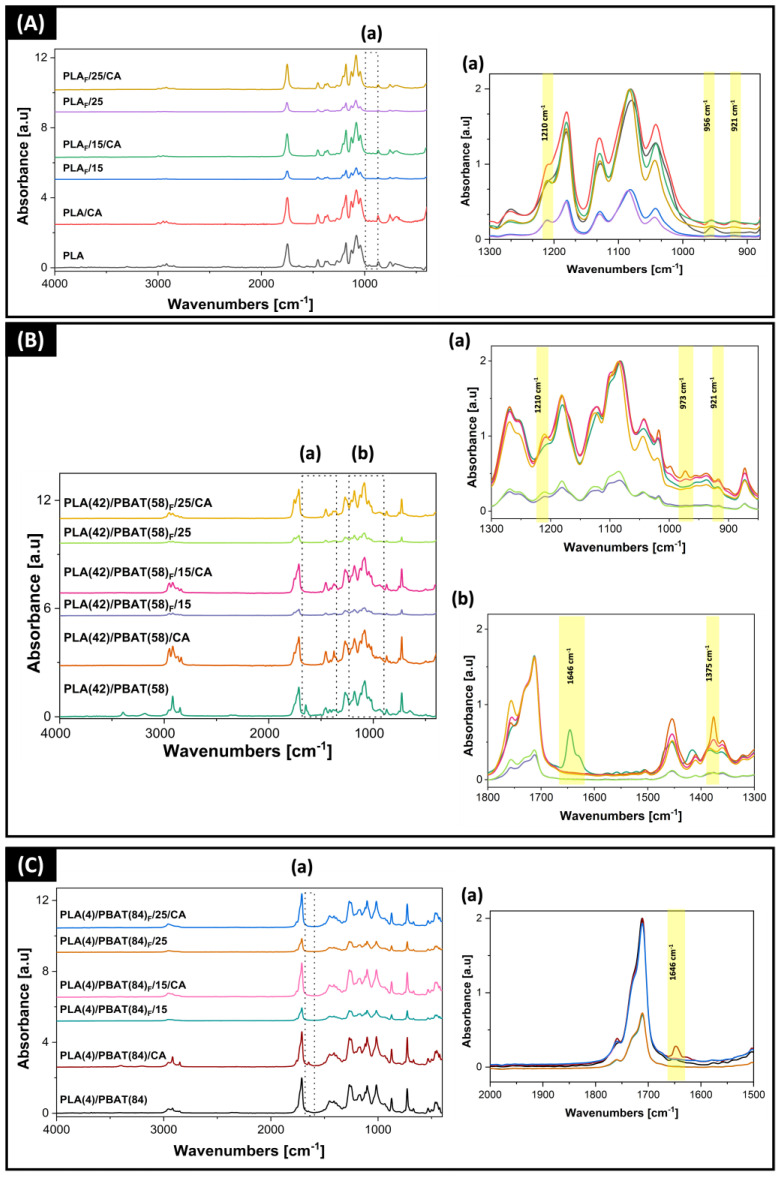
ATR-FTIR spectra of the obtained materials (films and foams) with and without the presence of active ingredient: (**A**) PLA and (**a**) zoom of the 1300 to 900 cm^−1^ region; (**B**) PLA(42)/PBAT(58) with (**a**) and (**b**) zoom of the 1300 to 900 cm^−1^ and 1800 to 1300 cm^−1^ regions, respectively; (**C**) PLA(4)/PBAT(84) and (**a**) zoom of the spectrum between 2000 and 1500 cm^−1^.

Figure 4A shows the spectra of neat PLA-impregnated films and foams. PLA samples presented their characteristic peaks, such as the carbonyl group C=O at 1747 cm^−1^, weak bands at 1453 and 1380 cm^−1^ assigned to the stretching of the methyl groups (CH_3_) [69,70], and peaks at 1180 and 1079 cm^−1^ associated with the asymmetric C-O-C and C-O vibrations [71]. Finally, the peaks at 867 cm^−1^ and 754 cm^−1^ were assigned to the C-C bond stretching attributed to the amorphous and crystalline phases of PLA [35,72].

To analyze the structural changes in the blends used with different proportions of PLA and PBAT, both for the impregnated films and the foams developed, the spectra corresponding to these materials are shown in Figure 4B,C.

In the case of films formed by blends (Figure 4B,C), considering that the chemical structure of PBAT is similar to PLA, the most important feature was the presence of phenyl rings at 1015 cm^−1^ in the PLA(4)/PBAT(84) blend [42,73], in addition to the 1712 cm^−1^ band corresponding to the C=O stretching of the carbonyl and ester groups in both blends, together with the 1267 cm^−1^ band assigned to the stretching of the C-O ester groups. Finally, a strong signal at 727 cm^−1^ corresponds to the stretching of the CH_2_ groups [73], while the signal at 873 cm^−1^ is assigned to the amorphous phase of PLA. Three weak absorption bands at 1209 cm^−1^, 956 cm^−1^, and 920 cm^−1^ (Figure 4(Aa,Ba)) were observed in samples of PLA and PLA(42)/PBAT(58) with CA (films and foams). These bands are due to the vibration of the C-O-C group caused by the asymmetric vibration of CH with respect to the amorphous fraction of PLA (which decreases in the presence of CA) and the α-crystalline phase, respectively [41,74,75]. In the case of PLA, although the intensity of the band around 1209 and 921 cm^−1^ was weak, the intensity of the peak increased with the addition of CA to the polymeric matrix. This was in contrast to the 956 cm^−1^ band, which decreased in intensity as the 921 cm^−1^ band increased.

On the other hand, the PLA(42)/PBAT(58) samples incorporating CA only showed an increase in intensity in the 1209 cm^−1^ and 921 cm^−1^ bands. It should be noted that these signals were related by Carrasco et al. [67] with PLA morphology. The appearance and variation in the intensity of these bands could indicate that the presence of CA favors the crystallization of PLA since, for both polymers, the higher intensity of both bands was observed in the case of impregnated films. This would confirm the slight increase in the degree of crystallinity of PLA and the PLA(42)/PBAT(58) blend observed in the DSC analysis of these samples.

On the other hand, the films with and without CA of PLA(42)/PBAT(58) and PLA(4)/PBAT(84) showed changes in the band located at 1646 cm^−1^ (Figure 4(Bb,Ca)). These changes could be related to the CO_2_ impregnation process since CO_2_ produces a swelling effect that generates mobility of the polymer chains and can subsequently reduce the penetration of the IR beam. This could indicate that the swelling effect of the polymeric matrix may be partially irreversible and/or that the ordering of the polymer chains is modified after the system is depressurized [76]. 

Finally, the PLA(42)/PBAT(58) film and the foam samples with the incorporation of CA showed two characteristic bands at 1375 and 973 cm^−1^ that are associated with phenolic hydroxyls (Figure 4(Ba,Bb)) [77] and the bending of the C-H group out of plane [78], respectively. It should be noted that this was the material with the highest concentration of impregnated CA.

### 3.5. Morphological Analysis of the Obtained Materials 

**Figure 5:**
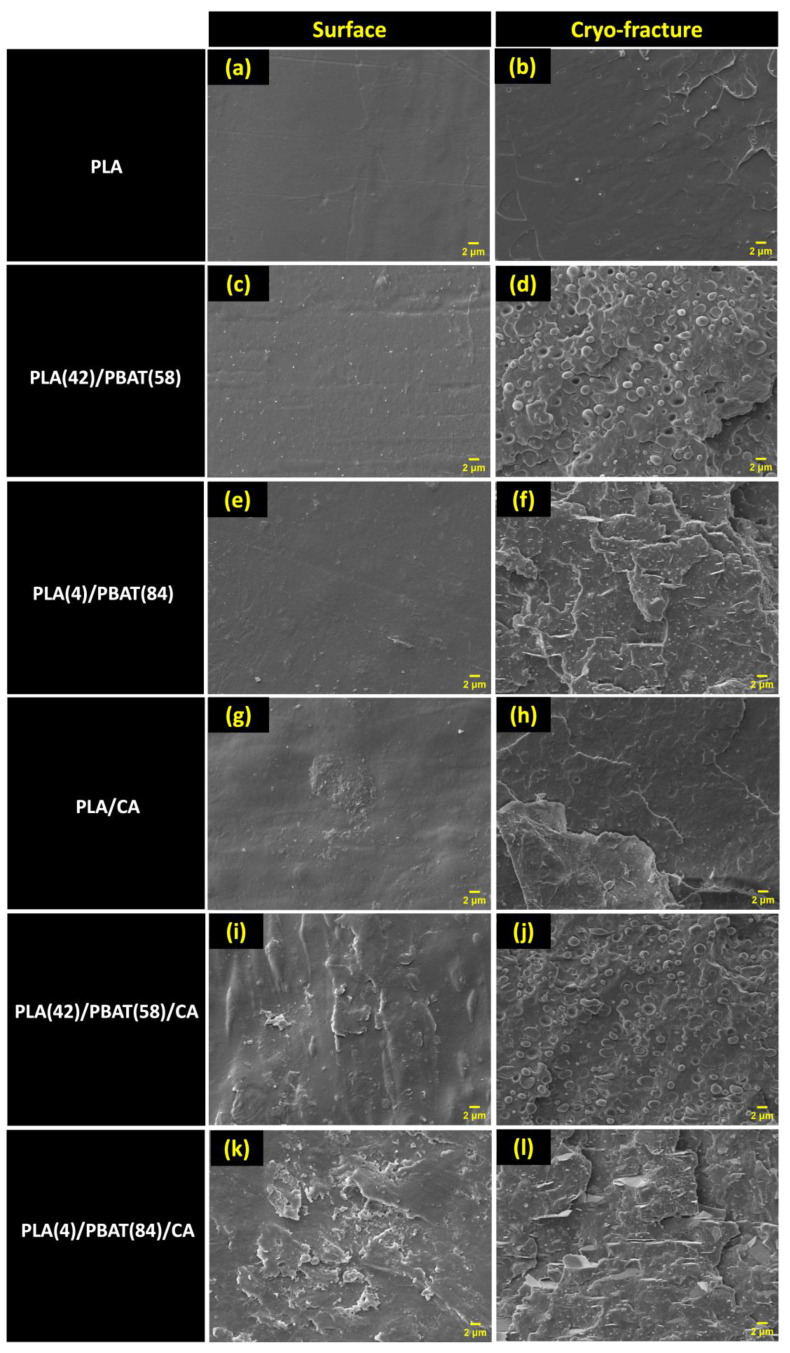
SEM micrographs at 5.00 kx. magnification of the films with and without active compound. Column (**a**,**c**,**e**,**g**,**i**,**k**) show the surface images of the films. While in (**b**,**d**,**f**,**h**,**j**,**l**) correspond to the surface of the cryofractured films.

The films with the highest CA incorporation for each material were observed by scanning electron microscopy (Figure 5). For comparison, untreated PLA, PLA(42)/PBAT(58) and PLA(4)/PBAT(84) films were also assayed.

The neat PLA showed the regular surface typical of this polymer (Figure 5a,b) [79], while very similar behavior was observed in the PLA(42)/PBAT(58) (Figure 5c,d) and PLA(4)/PBAT(84) (Figure 5e,f). The cryo-fractured surface of PLA (Figure 5a,b) exhibits the typical smooth and regular surface of amorphous polymers characteristic of PLA [79], in contrast to the micrographs of the cryo-fractured surface of PLA/PBAT blends, which showed two phases, the hard and the soft phases, corresponding to the PLA and PBAT matrix, respectively. This is due to the low compatibility between the two polymeric matrices (PLA and PBAT), which results similar to studies carried out by other authors [18,80,81] and is in agreement with the results obtained from the DSC analysis of the samples. The PBAT phase can be evidently seen in the form of spherical particles imbibed in the PLA matrix in the PLA(42)/PBAT(58) sample, and a slight amount of small voids are also evident (Figure 5c,d). The spherical particles are less pronounced in the case of the PLA(4)/PBAT(84) sample, and there were no voids in this sample due to the lower amount of PBAT; however, it still shows a rougher fracture behavior. According to Correa-Pacheco et al. [81], the dispersed phase is the minority phase of PBAT that is homogeneously distributed throughout the polymeric matrix, maintaining some interfacial tension, which seems to be more homogeneous when a lower amount of PBAT is in the formulation. Finally, for the CA-impregnated samples (Figure 5), it was observed that for all three polymeric matrices, i.e., PLA (Figure 5g,h) and the PLA/PBAT blends of PLA(42)/PBAT(58) (Figure 5i,j) and PLA(4)/PBAT(84) (Figure 5k,l), changes were mainly observed in the film surface which becomes somewhat rougher as a consequence of the presence of CA. Similarly, in the case of cryo-fractured samples, an increase in the rougher behavior is observed due to the presence of CA, which is particularly evident in PLA/CA (Figure 5g,h) since the neat PLA film sample was completely regular (Figure 5a,b) and the change is biggest due to the PLA/CA surface not having a plain smoothness. For PLA(42)/PBAT(58)/CA (Figure 5i,j), the spherical PBAT particles imbibed in the PLA polymeric matrix are also observed, but the small voids observed in its PLA(4)/PBAT(84) counterpart (Figure 5k,l) completely disappeared suggesting that CA is somewhat improving the compatibility between both polymeric matrices. For PLA(4)/PBAT(84)/CA (Figure 5k,l), the cryo-fracture surface is very similar to that of its PLA(4)/PBAT(84) (Figure 5e,f) counterpart.

Considering that the morphology of the foams is characterized by size, shape, cell density, variability of cell size, and apparent density, microstructural analyses of the different foams obtained were carried out using a scanning electron microscope (SEM). The aim was to evaluate the effect of pressure on the morphology of the PLA/PBAT blends as well as the CA impregnation in comparison to neat PLA foams to establish the optimal processing conditions for PLA/PBAT blends foams. Table 5 shows the cell size (d), bulk density (ρf), cell density (NC), and expansion ratio (ER) of PLA foams and PLA/PBAT blends with and without active agents.

From Table 5, it can be seen that both PLA and blends produce microcellular foams [82], as their sizes are between 1 and 100 µm. In addition, the increase in pressure caused a decrease in pore diameter for all foams. At the same time, the density of the foams increased when a higher proportion of PBAT was used in the blend. This can be attributed to the heterogeneous nucleation effect of PBAT [65]. As the pressure increases, the solubility of scCO_2_ improves, which favors the dissolution of the polymer in the supercritical phase, resulting in a higher cell density and smaller diameter of the polymer foams [83]. These trends are in agreement with the results of Hu and coworkers [84], who developed PBAT/PBS blend foams with different PBAT/PBS ratios.

**Figure 6:**
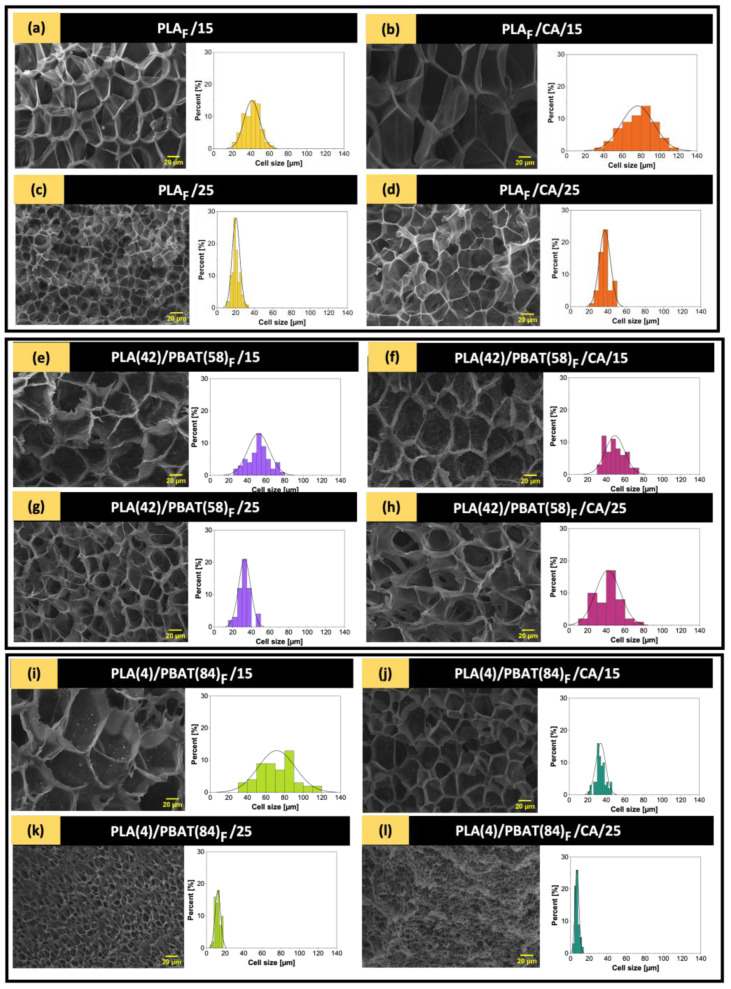
SEM micrographs of PLA foams and PLA/PBAT blends cryo-fractured at 15 and 25 MPa with and without active compound.

In the case of active foams, the nucleating effect of the presence of CA can be observed in the foams processed at 15 MPa, which would confirm what was reported in the DSC analyses. Moreover, according to the results obtained in the quantification of CA in the developed foams, the foams processed at 15 MPa maintained a higher amount of impregnated CA compared to the foams impregnated at 25 MPa. Figure 6 shows the effects of the presence of PBAT in the PLA-based blends, the addition of CA, and the pressure variation in the foaming process on the cell morphologies of the PLA and PLA/PBAT foams obtained from the SEM micrographs of the cross-sections of the cryo-fractured samples, as well as the cell diameter distributions based on the Gaussian distribution.

Figure 6 shows that the presence of PBAT contributed to cell wall thickening, in addition to obtaining more uniform and well-distributed cell sizes, which in turn were smaller with increasing system pressure and amount of PBAT in the blend and, thus, the foams processed at 25 MPa, PLA(4)/PBAT(84)_F_ (Figure 6k) and PLA(4)/PBAT(84)/CA_F_ (Figure 6l), were those with smallest cells and showed the most uniform and well-distributed cell sizes. This can be attributed to PBAT acting as a nucleating agent in PLA, inducing crystal formation and increasing PLA melt strength [47,85], which is also consistent with Shi et al., who investigated the influence of PBAT and CaCO_3_ on PLA crystallization behavior [62]. 

Regarding the results shown in Figure 6 and Table 5, it can be observed that the PLA and PBAT samples foamed with and without caffeic acid show different changes in cell size. The foaming process of PLA in the presence of caffeic acid shows a significant increase in the cell size from 41.05 µm in neat PLA at 15 MPa (Figure 6a) to 76.22 µm in PLA/CA at 15 MPa system (Figure 6b). Meanwhile, the behavior of PBAT (84 wt%) is the opposite of this, as the cell size is significantly reduced in samples foamed in the presence of caffeic acid (Figure 6j,l). Moreover, in the samples with balanced amounts of each polymer PLA(42)/PBAT(58)_F_, this change in the cell size can be considered negligible (Figure 6f,h).

It should be noted that the foam samples containing CA had already been treated with scCO_2_ to impregnate the active compound. Thus, an effect of the prior supercritical impregnation could also be responsible for this change in the cell size during the foaming process.

In this context, the sequential supercritical processing to load CA to prepare the polymeric foam, as well as the chemical nature of the CA and the difference in structure of the two polymers, which have different values of thermal properties, could generate different expansions during the cell formation in the foaming process as a result of their different mechanical properties combined with the different desorption rates of CO_2_ from the samples and through their structures.

To understand the effects of CA, polymer structure, and prior supercritical processing on the cell size of these foams, further experiments should be designed and performed to isolate the effect of these variables.

Considering the above, the presence of both amorphous and crystalline structures influences the formation of PLA foams, which is why the presence of PBAT [65] leads to modifications in the cell size, density, and expansion coefficient of the PLA/PBAT-based foams, influencing their crystalline behavior, as reported in the DSC analysis.

### 3.6. Antioxidant Activity of Obtained Films and Foams

**Figure 7:**
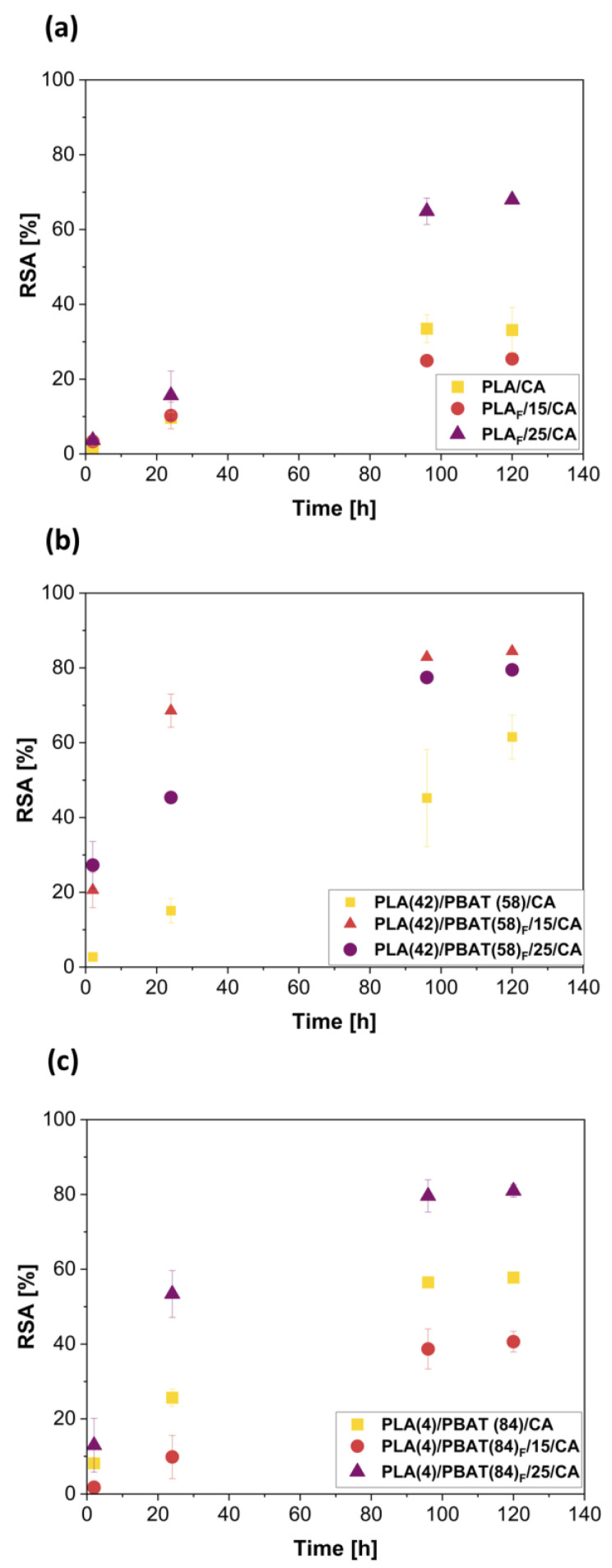
Antioxidant activity of films and foams impregnated with CA, expressed as inhibition percentage. (**a**) PLA; (**b**) PLA(42)/PBAT(58); (**c**) PLA(4)/PBAT(84).

Figure 7 shows the antioxidant behavior of CA developed from PLA-CA (Figure 7a) and PLA-PBAT-CA (Figure 7b,c) materials in direct contact with DPPH solution. The inhibition of the DPPH radical is expressed as a percentage after 2, 12, 24, 196, and 120 h.

In the PLA/CA-based materials, it was observed that at the beginning, the antioxidant activity was very similar in all the PLA/CA-based materials up to 24 h, whereas a significantly higher antioxidant activity was observed for the PLA/CA/25 foam at 96 h, reaching the steady state which shows about 70% of the RSA. From the beginning until 24 h, the RSA activity is due to the CA on the surface, which is in direct contact with the DPPH solution. Meanwhile, after 96 h, the higher antioxidant activity of PLA/CA/25 foam can be attributed to the well-dispersed CA in this system as a consequence of scCO_2_.

Although the film of PLA(42)/PBAT(58)/CA possesses a higher amount of CA than the PLA(42)/PBAT(58)/CA-based foams, the highest antioxidant activity is observed in both foams due to the porous structure of the foam that allow higher contact with the DPPH solutions in the whole material, while the films only expose the surface. 

The PLA(4)/PBAT(84)/CA foam processed at 25 MPa showed a higher antioxidant activity than the foam processed at 15 MPa, even though it has a lower amount of CA. This behavior could be attributed to the fact that at 25 MPa, the polymer matrix is plasticized due to the high amount of CO_2_, which increases the mobility of the polymer chains and facilitates the release of the CA. In addition, a better interaction with the DPPH solution is possible due to the smaller porous structure of this material.

In fact, CA has been chosen as the antioxidant compound in this work, as it is known to be very effective in scavenging free radicals since its structure presents very active -OH groups in the phenolic rings, as it has the second hydroxyl group in the ortho position, which favor the conjugation of the double C=C bonds, while the carboxylic group is also involved in the conjugation [79,86]. It should be highlighted that despite the low amount of CA incorporated in the foamed structure, the obtained materials showed antioxidant activity in the range of other biopolyesters, such as electrospun PHB mats loaded with higher amounts of CA (i.e., 20 wt% of CA) [87]. These results show that the foamed structure is able to expose a higher surface area to the DPPH solution, especially when smaller cells were obtained, giving the material with antioxidant activity at the beginning of the test (2 h). This is a very interesting result, since it has been observed that in films blending PLA with another more crystalline polymer, an induction period is generated in the diffusion process due to the limited polymer chain mobility [88]. Then, the plasticizing effect of CO_2_ favors the release of CA, and this release reaches higher values in less crystalline foams (see Table 3). The results showed the potential of using foamed structures for the development of active packaging materials, since foams allow to obtain polymeric structures with good antioxidant activity with low amounts of active ingredients, from the initial production to the contact with food.

## 4. Conclusions

PLA polymeric foams and PLA-based blends have been developed with and without impregnated CA at different processing conditions. 

The use of ethanol as a co-solvent made it possible to obtain a higher amount of impregnated active compound, both in the PLA samples and in the blends that were analyzed. In the case of the PLA samples, the highest amount of impregnated CA was obtained at a pressure of 15 MPa and a depressurization rate of 1 MPa/min. For the blends analyzed, higher amounts of impregnated CA were obtained at 12 MPa but at different depressurization rates of 1 (MPa/min) and 0.1 (MPa/min) for PLA(42)/PBAT(58) and PLA(4)/PBAT(84), respectively.

DSC analyses revealed an increase in the degree of crystallinity of the CA-impregnated samples, suggesting the nucleating effect induced by the presence of the active agent and the presence of CaCO_3_ in the PLA(4)/PBAT(84) samples. On the other hand, SEM images for both films and foams showed significant differences due to the presence of PBAT and, in turn, to the low miscibility of this with PLA. The antioxidant effectiveness of the CA released from the materials to a DPPH solution showed that the foamed structure facilitates the release of the active compound, allowing the production of materials with antioxidant properties using low amounts of active compounds. Once in contact with food, the release of CA starts immediately, while further release can be tuned by controlling the crystallinity of the system by adding of different amounts of PBAT.

Finally, the results of this work contribute to the knowledge of the important parameters to be taken into account for the feasibility of the impregnation and foaming process of PLA/PBAT blends in one step.

## Figures and Tables

**Table 1:** Operational conditions selected for the supercritical impregnation of caffeic acid.

Sample	Pressure [MPa]	Depressurization Rate [MPa/min]	Co-Solvent [wt%]
PLA/CA	15	1	5
PLA(42)/PBAT(58)/CA	12	1	5
PLA(4)/PBAT(84)/CA	12	0.1	5

**Table 2:** The amount of caffeic acid (q_CA_) present after the foaming process expressed as % [mg caffeic acid/mg polymer].

Sample	Type	Pressure [MPa]	q_CA_ [mg Caffeic Acid/mg Polymer]
PLA/CA	Film	-	0.0035 ± 0.0005
PLA_F_/CA	Foam	15	0.0007 ± 0.0001
PLA_F_/CA	Foam	25	0.0007 ± 0.0003
PLA(42)/PBAT(58)/CA	Film	-	0.0055 ± 0.0004
PLA(42)/PBAT(58)_F_/CA	Foam	15	0.0032 ± 0.0001
PLA(42)/PBAT(58)_F_/CA	Foam	25	0.0021 ± 0.0003
PLA(4)/PBAT(84)/CA	Film	-	0.0026 ± 0.0003
PLA(4)/PBAT(84)_F_/CA	Foam	15	0.0016 ± 0.0002
PLA(4)/PBAT(84)_F_/CA	Foam	25	0.0011 ± 0.0001

**Table 3:** DSC thermal properties of PLA and PLA/PBAT films with and without CA impregnation.

Sample	T_g PBAT_ [°C]	T_g PLA_ [°C]	T_m PBAT_ [°C]	ΔH_m PBAT_ [J/g]	T_m PLA_ [°C]	ΔH_m PLA_ [J/g]	%X_c PLA_
PLA	N.D.	58.1 ± 0.2 ^d^	N.D.	N.D.	148.2 ± 0.2 ^b^	29.2 ± 0.4 ^a^	4.1 ± 0.2 ^a^
PLA(42)/PBAT(58)	−31.3 ± 0.4 ^c^	57.4 ± 1.1 ^c^	115.1 ± 0.1 ^a^	7.1 ± 0.4 ^a^	148.9 ± 0.2 ^a,b^	2.3 ± 0.1 ^b^	5.7 ± 0.2 ^a^
PLA(4)/PBAT(84)	−30.8 ± 2.5 ^d^	N.D.	122.7 ± 0.1 ^b^	4.4 ± 0.2 ^b^	145.1 ± 0.7 ^c^	1.3 ± 0.1 ^c^	35.8 ± 1.6 ^b^
PLA/CA	N.D.	59.7 ± 0.4 ^b^	N.D.	N.D.	149.6 ± 0.1 ^a^	29.4 ± 0.5 ^a^	4.8 ± 0.4 ^a^
PLA(42)/PBAT(58)/CA	−30.2 ± 0.7 ^a^	59.1 ± 0.1 ^a^	114.5 ± 0.1 ^c^	6.2 ± 0.2 ^c^	149.7 ± 0.1 ^a^	2.9 ± 0.1 ^b^	7.5 ± 0.3 ^c^
PLA(4)/PBAT(84)/CA	−29.6 ± 1.4 ^b^	N.D.	123.4 ± 0.6 ^d^	4.5 ± 0.1 ^d^	145.3 ± 0.5 ^c^	1.4 ± 0.1 ^c^	36.2 ± 0.2 ^b^

Lowercase letters ^a–d^ indicate significant differences between the values of each thermal parameter. N.D.: not detected.

**Table 4:** Thermal properties of PLA and blend foams obtained at different pressure conditions.

Sample	Pressure [MPa]	T_m_ PLA [°C]	ΔH_m_ [J/g] PLA	%X_c_
PLA_F_	15	153.54 ± 0.61 ^c,d,e^	46.05 ± 4.28 ^a^	49.19 ± 4.57 ^b^
PLA(42)/PBAT(58)_F_		152.45 ± 0.00 ^a,b^	9.87 ± 0.71 ^f^	25.09 ± 1.82 ^g^
PLA(4)/PBAT(84)_F_		150.90 ± 0.24 ^b,c^	1.95 ± 0.07 ^h^	52.08 ± 2.27 ^b^
PLA_F_	25	148.85 ± 0.41 ^c,d,e,f,g^	32.96 ± 0.35 ^b^	35.21 ± 0.37 ^c,d,e^
PLA(42)/PBAT(58)_F_		147.23 ± 0.53 ^g,h^	15.23 ± 2.30 ^e^	38.73 ± 5.85 ^c^
PLA(4)/PBAT(84)_F_		147.30 ± 0.20 ^g,h^	0.97 ± 0.00 ^h^	27.78 ± 2.64 ^f,g^
PLA_F_/CA	15	153.41 ± 0.34 ^b,c,d^	39.00 ± 1.65 ^b^	41.67 ± 1.77 ^c,d^
PLA(42)/PBAT(58)_F_/CA		153.30 ± 0.12 ^a^	11.45 ± 0.73 ^f^	29.11 ± 1.85 ^e,f,g^
PLA(4)/PBAT(84)_F_/CA		149.94 ± 0.32 ^c,d,e,f^	2.41 ± 0.03 ^h^	64.37 ± 1.13 ^a^
PLA_F_/CA	25	150.42 ± 1.59 ^c,d,e,f,g^	33.94 ± 1.55 ^b^	36.26 ± 1.65 ^c,d,e^
PLA(42)/PBAT(58)_F_/CA		147.12 ± 0.10 ^g,h^	15.59 ± 1.06 ^e^	39.66 ± 2.70 ^c^
PLA(4)/PBAT(84)_F_/CA		153.13 ± 0.38 ^a^	1.04 ± 0.01 ^h^	27.64 ± 0.19 ^f,g^

Lowercase letters ^a–h^ indicate significant differences between the values of each thermal parameter.

**Table 5:** Cell size (d), foam density (ρf), cell density (NC), and expansion ratio (ER).

Sample	Pressure (MPa)	d (µm)	ρ_f_ (kg/m^3^)	NC (×10^11^ cell/cm^3^)	ER
PLA_F_	15	41.05 ± 8.37	104.7 ± 4.77	1.29	9.13
PLA(42)/PBAT(58)_F_		52.00 ± 11.64	269.63 ± 1.49	0.50	3.32
PLA(4)/PBAT(84)_F_		72.87 ± 19.87	203.64 ± 8.67	0.21	5.00
PLA_F_	25	20.26 ± 4.10	120.39 ± 46.19	10.5	7.94
PLA(42)/PBAT(58)_F_		32.65 ± 6.44	124.23 ± 38.03	2.52	7.21
PLA(4)/PBAT(84) _F_		12.21 ± 2.99	295.53 ± 46.66	39	3.44
PLA_F_/CA	15	76.22 ± 17.62	125.24 ± 34.33	0.20	7.57
PLA(42)/PBAT(58)_F_/CA		49.17 ± 10.37	302.59 ± 39.57	0.57	3.08
PLA(4)/PBAT(84)_F_/CA		33.77 ± 5.32	237.84 ± 14.12	1.95	4.00
PLA_F_/CA	25	36.72 ± 6.01	83.83 ± 4.26	1.84	11.31
PLA(42)/PBAT(58)_F_/CA		41.69 ± 13.32	96.53 ± 8.29	1.24	9.66
PLA(4)/PBAT(84)_F_/CA		6.91 ± 2.13	327.86 ± 8.54	198	2.90

## Data Availability

The data presented in this study are available upon request from the corresponding authors (due to privacy).
